# Structural characteristics of BtKY72 RBD bound to bat ACE2 reveal multiple key residues affecting ACE2 usage of sarbecoviruses

**DOI:** 10.1128/mbio.01404-24

**Published:** 2024-07-31

**Authors:** Chao Su, Juanhua He, Liang Wang, Yu Hu, Jian Cao, Bin Bai, Jianxun Qi, George Fu Gao, Mengsu Yang, Qihui Wang

**Affiliations:** 1 CAS Key Laboratory of Pathogen Microbiology and Immunology, Institute of Microbiology, Chinese Academy of Sciences (CAS), Beijing, China; 2 Department of Biomedical Sciences, City University of Hong Kong, Hong Kong SAR, China; 3 Institute of Pediatrics, Shenzhen Children's Hospital, Shenzhen, Guangdong, China; 4 CAS Key Laboratory of Pathogen Microbiology and Immunology, Institute of Microbiology, Center for Influenza Research and Early-warning (CASCIRE), CAS-TWAS Center of Excellence for Emerging Infectious Diseases (CEEID), Chinese Academy of Sciences, Beijing, China; 5 School of Life Sciences, Division of Life Sciences and Medicine, University of Science and Technology of China, Hefei, Anhui, China; 6 University of the Chinese Academy of Sciences, Beijing, China; Washington University in St. Louis School of Medicine, St. Louis, Missouri, USA

**Keywords:** BtKY72, BM48-31, SARS-CoV-2, ACE2, sarbecovirus

## Abstract

Two different sarbecoviruses, severe acute respiratory syndrome coronavirus (SARS-CoV) and SARS-CoV-2, have caused serious challenges to public health. Certain sarbecoviruses utilize angiotensin-converting enzyme 2 (ACE2) as their cellular receptor, whereas some do not, speculatively due to the two deletions in their receptor-binding domain (RBD). However, it remains unclear whether sarbecoviruses with one deletion in the RBD can still bind to ACE2. Here, we showed that two phylogenetically related sarbecoviruses with one deletion, BtKY72 and BM48-31, displayed a different ACE2-usage range. The cryo-electron microscopy structure of BtKY72 RBD bound to bat ACE2 identified a key residue important for the interaction between RBD and ACE2. In addition, we demonstrated that the mutations involving four types of core residues enabled the sarbecoviruses with deletion(s) to bind to human ACE2 (hACE2) and broadened the ACE2 usage of SARS-CoV-2. Our findings help predict the potential hACE2-binding ability to emerge sarbecoviruses and develop pan-sarbecovirus therapeutic agents.

**IMPORTANCE:**

Many sarbecoviruses, including severe acute respiratory syndrome coronavirus 2 (SARS-CoV-2), possess the ability to bind to receptor angiotensin-converting enzyme 2 (ACE2) through their receptor-binding domain (RBD). However, certain sarbecoviruses with deletion(s) in the RBD lack this capability. In this study, we investigated two closely related short-deletion sarbecoviruses, BtKY72 and BM48-31, and revealed that BtKY72 exhibited a broader ACE2-binding spectrum compared to BM48-31. Structural analysis of the BtKY72 RBD-bat ACE2 complex identifies a critical residue at position 493 contributing to these differences. Furthermore, we demonstrated that the mutations involving four core residues in the RBD enabled the sarbecoviruses with deletion(s) to bind to human ACE2 and expanded the ACE2 usage spectra of SARS-CoV-2. These findings offer crucial insights for accurately predicting the potential threat of newly emerging sarbecoviruses to human health.

## INTRODUCTION

Two members of the sarbecovirus subgenus, severe acute respiratory syndrome coronavirus (SARS-CoV) and SARS-CoV-2, are well known. SARS-CoV has caused >8,000 confirmed cases worldwide, with a fatality rate of approximately 10% (1). More than 767 million people have been infected with SARS-CoV-2, with more than six million deaths (covid19.who.int) and massive economic losses until July 2023. Sarbecoviruses also comprise a variety of bat-origin viruses (2–17) and a number of pangolin-origin viruses (18, 19). Many of these are considered to be at high risk of potential emergence. WIV1 (11) and RsSHC014 (20) can replicate efficiently in primary human airway epithelial cells. With the development of next-generation sequencing (NGS) technology, a surge in sarbecoviruses has been detected in wild animal populations globally (2–9, 18, 19, 21). However, minimal additional research has been conducted on these sarbecoviruses beyond the phylogenetic analysis of genomes. This lack of exploration leaves us uncertain about whether these viruses have the potential to infect humans.

The ability of sarbecoviruses to enter host cells depends on the binding of the spike (S) protein to its receptor (22, 23). Angiotensin-converting enzyme 2 (ACE2) has been reported to be an entry receptor for many sarbecoviruses (9, 13, 14, 24–27). SARS-CoV, SARS-CoV-2 GD/1/2019, GX/P2V/2017, and RaTG13 were found to bind to ACE2 from many species, including humans, with varied binding affinities (28–30). Structural studies of the receptor-binding domain (RBD) protein bound to human ACE2 (hACE2) showed that four interfacial segments in these RBDs referred to as regions 1, region 2, region 3, and receptor binding ridge (4) (Fig. S1) are involved in binding to hACE2, with a similar binding mode (9, 27, 28, 31, 32), suggesting their close phylogenetical relationship. Notably, many sarbecovirus RBDs exhibit deletions in either of the region 2, the receptor binding ridge, or both regions. We named them short-deletion, long-deletion, and two-deletion, respectively (Fig. S1).

The molecular mechanism of binding to the receptor ACE2 has primarily been studied in sarbecoviruses with full-length RBD. Many short-deletion and two-deletion sarbecoviruses, unlike the full-length sarbecoviruses mentioned above, lack hACE2-binding capacity (33, 34). It has been reported that the SARS-CoV-2 short-deletion RBD substantially reduced the binding affinity with hACE2 (32). To date, there have been no reports of two-deletion sarbecovirus RBD binding to any ACE2 (34). Interestingly, some short-deletion sarbecoviruses, like BM48-31, have shown limited binding ability to ACE2 receptors (34, 35), while others such as BtKY72 and RaTG15 exhibit ACE2-binding capability, such as to intermediate horseshoe bat ACE2, but not to hACE2 (2, 34). Moreover, a recently discovered short-deletion sarbecovirus, Khosta-2, was demonstrated to possess hACE2-binding capacity (25, 34). The observation that BtKY72, BM48-31, and Khosta-2 are phylogenetically closely related based on RBD sequences (6, 34) (Fig. S1) suggests that an evolutionary analysis cannot explain the variation in their ability to use hACE2 as a receptor. Thus, there is an urgent need to investigate the binding mechanism of short-deletion sarbecovirus RBD with ACE2 to predict the ACE2-binding ability of future newly discovered short-deletion sarbecoviruses.

Here, through the cryogenic electron microscopy (cryo-EM) structure of BtKY72 RBD interacting with lander’s horseshoe bat ACE2 (laACE2) and the sequence alignment of ACE2s, we revealed that the residue K493 contributes to the broader ACE2-binding spectra of BtKY72 RBD than BM48-31. Moreover, we identified four types of core amino acid residues in sarbecovirus RBDs and found that these residues help confer sarbecoviruses with deletion(s) in RBD the ability to bind to hACE2. In addition, the extra acquisition of the core residues expanded the ACE2 usage spectra of SARS-CoV-2. Taken together, these data provide useful information for predicting the hACE2-binding ability of newly emerging sarbecoviruses.

## RESULTS

### BtKY72 RBD bound to ACE2 orthologs more broadly than BM48-31 RBD

**Fig 1:**
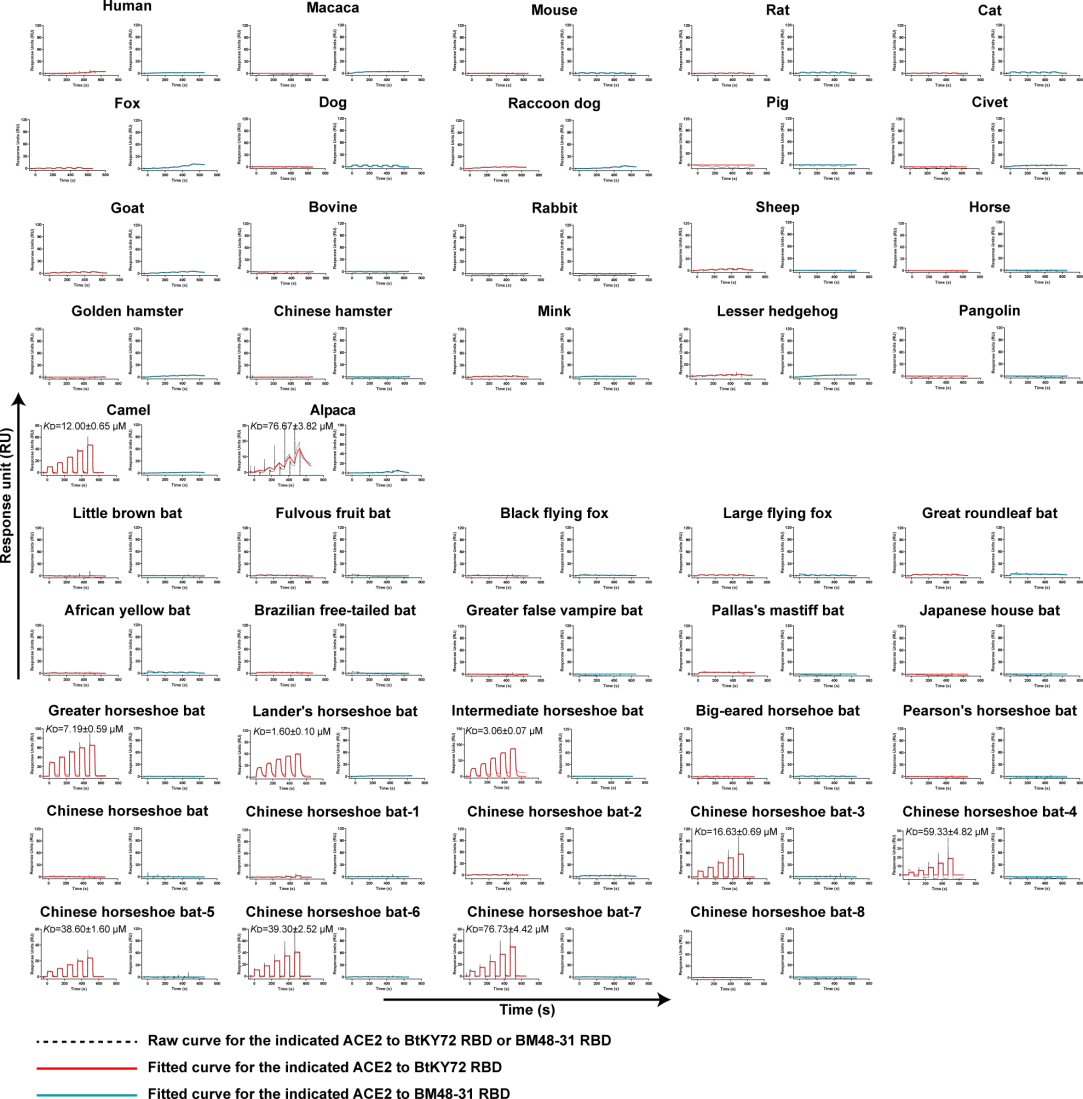
Binding affinity of BtKY72 RBD and BM48-31 RBDs for ACE2 orthologs, characterized by SPR. Mouse Fc (mFc)-fused ACE2 orthologs in cell supernatants were captured on CM5 chips through pre-immobilized anti-mFc antibodies. Serially diluted BtKY72 RBD and BM48-31 RBD were flowed over the chip to test the response unit, respectively. Representative results are shown from three independent experiments. *K*_D_ values in the pictures are shown as means ± SEM of three independent replicates (*n* = 3).

Phylogenetic analysis based on 106 sarbecovirus RBD sequences suggested that the majority of short-deletion RBDs were in clade 3 and the remainder were in clade 1b (34) (Fig. S1). Interestingly, all of the sarbecoviruses found in Europe and Africa were short-deletion. Thus, we selected BM48-31and BtKY72, which were found in Europe (10) and Africa (16), respectively, and used surface plasmon resonance (SPR) assays to test their binding to 46 different ACE2 orthologs from primate, rodent, carnivora, perissodactyla, artiodactyla, lagomorpha, erinaceomorpha, pholidota, and chiroptera orders. The SPR results revealed that BM48-31 RBD did not bind to any ACE2 orthologs tested, including hACE2 (Fig. 1). *Rhinolophus blasii* bats, which are the identified BM48-31 host (10), were not included in the tested species, due to the lack of sequence information for the ACE2 ortholog. However, BtKY72, which was identified from a rectal swab of a horseshoe bat in Kenya (16), binds to the lander’s horseshoe bat ACE2 with a *K*_D_ of 1.6 µM (Fig. 1), the highest binding affinity of all species studied. ACE2s from other horseshoe bat, such as the greater horseshoe bat (*Rhinolophus ferrumequinum*), intermediate horseshoe bat (*Rhinolophus affinis*), and Chinese horseshoe bat (*Rhinolophus sinicus*)−3/4/5/6/7, were also observed to interact with BtKY72 RBD, with binding affinities ranging from 3.06 μM to 76.73 µM (Fig. 1). Interestingly, BtKY72 RBD was able to bind to camel and alpaca ACE2s, but not to hACE2 (Fig. 1). Overall, as a short-deletion RBD, BtKY72 RBD displayed a broader ACE2 binding range than BM48-31 RBD.

### The complex structure of laACE2 bound to BtKY72 RBD

**Fig 2:**
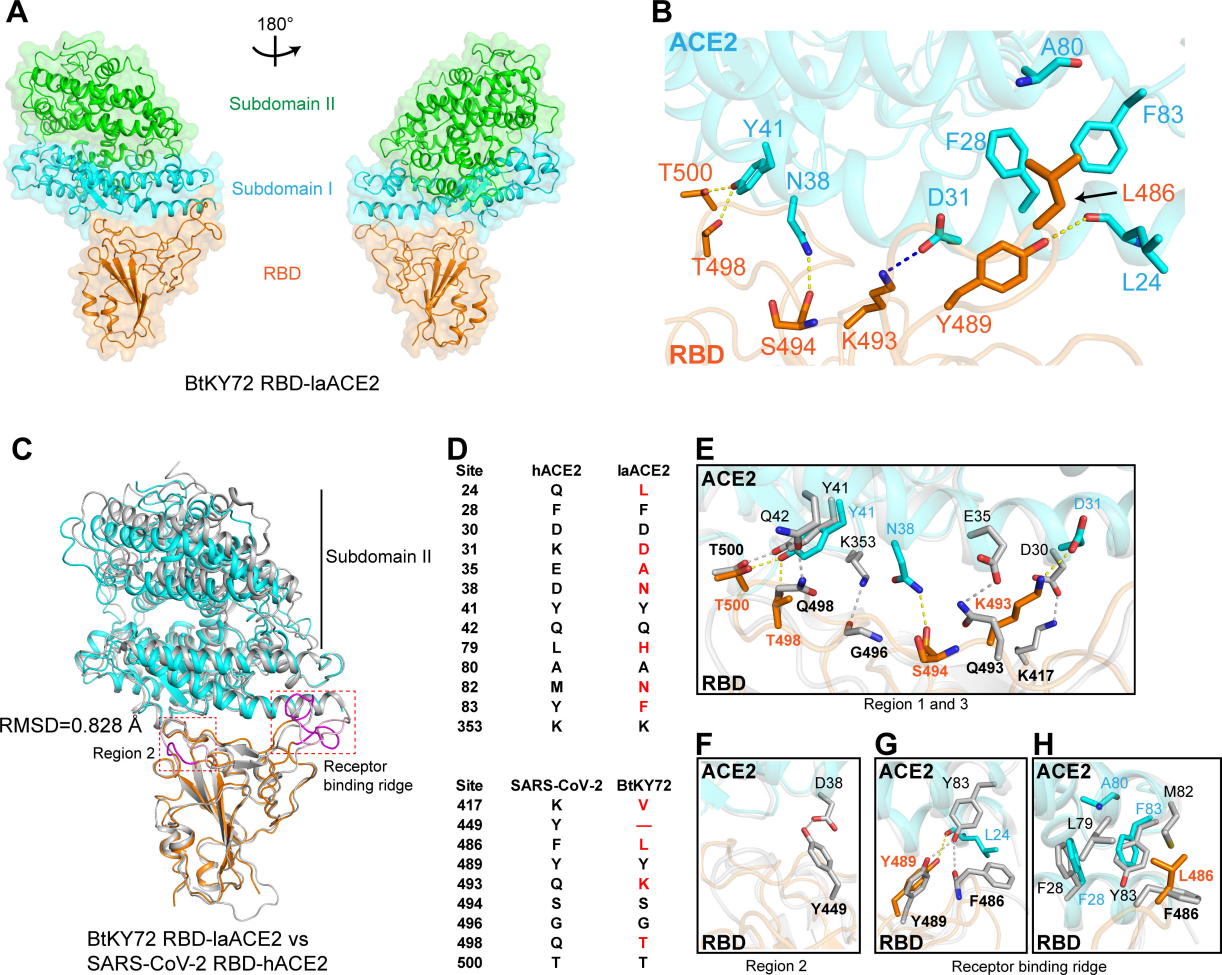
Complex structure of BtKY72 RBD bound to laACE2. (**A**) Cartoon structural representation of BtKY72 RBD-laACE2 complex. Subdomain I (S19–Q102, N290–N397, and P514–E430) and subdomain II (S103–P289, E398–T414, and D431–D615) of laACE2 based on hACE2 subdomain I and II (36) are colored in cyan and green, respectively. (**B**) Detailed interactions between BtKY72 RBD and laACE2. Key contact residues are shown as stick structures and labeled. Hydrogen bond interactions were analyzed at a cutoff of 3.5 Å and colored in yellow. The salt bridge is colored in blue. (**C**) Structural comparison of BtKY72 RBD-laACE2 and SARS-CoV-2 RBD-hACE2 (PDB: 7KMB). RMSD, region 2, and receptor binding ridge are shown. (**D**) Key amino acid residues that are involved in forming hydrogen bonds, salt bridges or hydrophobic interaction between BtKY72 RBD and laACE2 or SARS-CoV-2 RBD and hACE2. The number of RBD sites is based on the SARS-CoV-2 RBD sequence. Red residues indicate the laACE2 or BtKY72 RBD residues that are different from hACE2 or SARS-CoV-2. (**E–G**) Detailed comparison of hydrogen bonds and salt bridges in regions 1 and 3 (**E**), region 2 (**F**), and receptor binding ridge (**G**). SARS-CoV-2 RBD-hACE2 is colored in gray. BtKY72 RBD and laACE2 are colored in orange and cyan, respectively. (**H**) Detailed comparison of hydrophobic interaction in receptor binding ridge.

To reveal the molecular basis of BtKY72 RBD binding to ACE2, we prepared a protein complex comprised of BtKY72 RBD and laACE2 for cryo-EM. The cryo-EM complex structure was solved to 3.2 Å resolution. Similar to hACE2 (36), laACE2 is divided into two subdomains, subdomain I and subdomain II, and binds to BtKY72 RBD through its subdomain I (Fig. 2A). The key residues involved in complex formation were identified for further analyses. We renumbered all RBD sequences used in this study based on the SARS-CoV-2 RBD sequence. Residues located in the van der Waals interaction distance (4.5 Å cutoff) between the RBD and ACE2 were selected (Table S1). Among these, a series of hydrophilic residues are involved in the formation of hydrogen bonds and salt bridges. These polar contacts included laACE2 L24 interacting with BtKY72 RBD Y489, D31 interacting with K493, N38 interacting with S494, and Y41 with T498 and T500 (Fig. 2B). Moreover, BtKY72 RBD L486 formed interactions with the hydrophobic region constituted by laACE2 L24, F28, A80, and F83 (Fig. 2B). Taken together, virus-receptor engagement is dominated by polar contacts and hydrophobic interactions.

Considering that the affinity of full-length SARS-CoV-2 RBD for hACE2 could be as high as ~20 nM (37), which is much higher than the affinity between BtKY72 RBD and laACE2, we compared the complex structures of BtKY72 RBD-laACE2 and SARS-CoV-2 RBD-hACE2 (PDB: 7KMB). These two structures were superimposed based on their RBD moiety. Their overall structures were similar, with a root mean square deviation (RMSD) of 0.828 Å for 148 equivalent RBD Cα atoms, although subdomain II of ACE2s displayed flexibility (Fig. 2C). However, approximately half of the key residues involved in RBD-ACE2 interactions were different between the two complexes (Fig. 2D). SARS-CoV-2 and BtKY72 RBDs had approximately equal numbers of hydrogen bonds and salt bridges with ACE2 in regions 1 and 3 (hydrogen bond: 4 vs 3; salt bridge: 1 vs 1) (Fig. 2E), whereas these numbers were significantly different in regions 2 and receptor binding ridge (hydrogen bond: 3 vs 1). In detail, the deletion of BtKY72 RBD region 2 resulted in the formation of a shorter loop that was unable to engage laACE2, leading to a loss of interaction with laACE2 (Fig. 2C). On the other hand, an extended loop in the SARS-CoV-2 RBD formed one more hydrogen bond with hACE2 (Fig. 2G). Moreover, the flexible loops formed in the receptor binding ridge were not well superimposed in the two structures (Fig. 2C), resulting in one hydrogen bond difference between them (Fig. 2G) and the weaker hydrophobic interaction caused by BtKY72 RBD L486 than SARS-CoV-2 RBD F486 with ACE2 (Fig. 2H). In conclusion, fewer interactions involving regions 2 and receptor binding ridge lead to a lower affinity between BtKY72 RBD and laACE2 than between SARS-CoV-2 RBD and hACE2.

### Identification of key residue important for BtKY72 RBD binding to ACE2 orthologs

**Fig 3:**
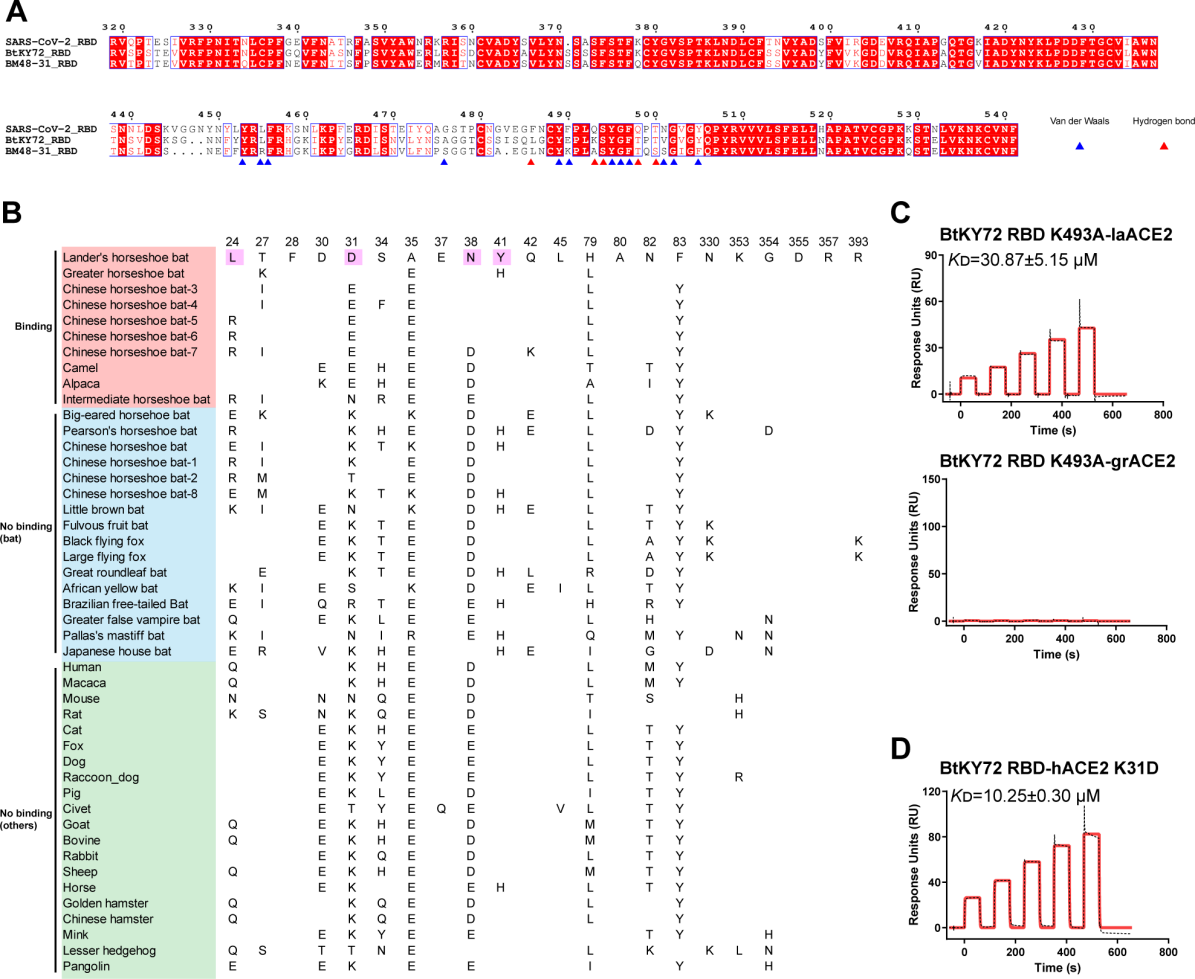
Residue K493 is key for BtKY72 RBD binding to ACE2 orthologs. (**A**) Sequence alignment of SARS-CoV-2, BtKY72, and BM48-31 RBD proteins. Blue and red triangles indicate the residues in BtKY72 RBD that are involved in binding to laACE2 by van der Waals and hydrogen bonds, respectively. (**B**) Key BtKY72 RBD-binding residues among ACE2 orthologs. The magenta rectangle indicates the residues of laACE2 forming a hydrogen bond or salt bridge with BtKY72 RBD. Blank letters indicate the same residue with laACE2. (**C and D**) Similar to Fig. 1, laACE2 and grACE2 (**C**), hACE2 K31D (**D**) proteins were captured on a CM5 chip, and various concentrations of BtKY72 K493A (**C**) and BtKY72 RBD (**D**) were used to test the binding affinity to ACE2. *K*_D_ value is shown in the pictures as means ± SEM of three independent replicates (*n* = 3).

To elucidate the molecular mechanism underlying the broader ACE2 ortholog binding range of BtKY72 RBD than that of BM48-31 RBD, we compared the two RBD sequences. The residues in the BtKY72 RBD, which are involved in forming hydrogen bonds and hydrophobic interactions with laACE2, are relatively conserved in the BM48-31 RBD, including L486, S494, T498, and T500 (S500 in BM48-31) (Fig. 3A). However, the BtKY72 RBD residue K493 corresponds to the BM48-31 residue A493, which is unable to form a salt bridge with laACE2 D31 (Fig. 3A). Then, we analyzed laACE2 D31 counterparts of the remaining 46 ACE2s and found that ACE2s with the BtKY72 RBD-binding abilities contain negatively charged residue at position 31, including aspartic acid and glutamic acid. The exception is the intermediate horseshoe bat ACE2, which involves asparagine. While it is positively charged or non-charged in non-RBD-binding ACE2s, it cannot form a salt bridge with BtKY72 RBD K493 (Fig. 3B). Thus, we hypothesized that RBD K493 is the key residue to determine the interaction between BtKY72 RBD and ACE2 orthologs.

To verify this hypothesis, we constructed BtKY72 RBD K493A mutant to test its binding to laACE2 and greater horseshoe bat ACE2 (grACE2). Through SPR assay (Fig. 3C), BtKY72 RBD K493A mutant was observed to bind to laACE2 with a *K*_D_ of 30.87 µM, representing an approximately 20-fold reduction in the binding affinity compared to the prototype (PT) BtKY72 RBD (*K*_D_ = 1.6 µM). The mutant lost the binding ability with grACE2. Furthermore, we introduced D31 to hACE2, which possesses K31, to construct a hACE2 K31D mutant. The SPR results showed that hACE2 K31D acquired the binding to BtKY72 RBD with a *K*_D_ of 10.25 µM (Fig. 3D). These results supported that residue K493 plays a key role in the interaction with ACE2.

### Identification of multiple single-point mutations enables BtKY72 RBD to bind to hACE2

**Fig 4:**
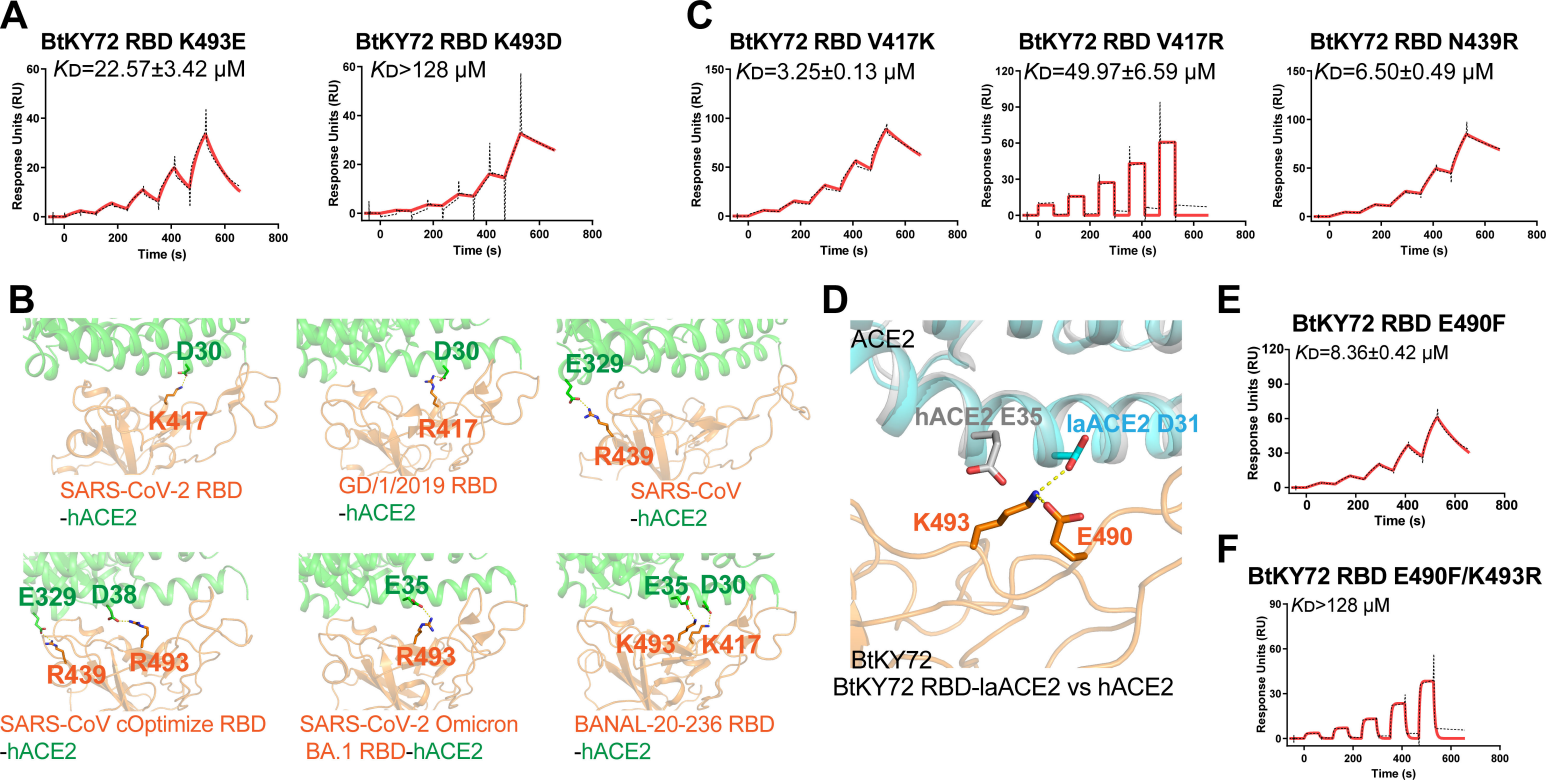
Multiple single-point mutations enable BtKY72 RBD to bind to hACE2. (**A, C, E, and F**) Similar to Fig. 1, hACE2 proteins were captured on a CM5 chip. BtKY72 RBD K493E and K493D (**A**), V417K, V417R, and N439R (**C**), E493F (**E**), and E490F/K493R (**F**) mutants were used to measure the binding affinity to hACE2. *K*_D_ values are shown in the pictures as means ± SEM of three independent replicates (*n* = 3). (**B**) Cartoon structural representation of SARS-CoV-2 RBD-hACE2 (PDB: 6LZG), GD/1/2019 RBD-hACE2 (PDB: 7DDO), SARS-CoV RBD-hACE2 (PDB: 2AJF), SARS-CoV cOptimize RBD-hACE2 (PDB: 3SCJ), SARS-CoV-2 Omicron BA.1 RBD-hACE2 (PDB: 7WBP), and BANAL-20–236 RBD-hACE2 (PDB: 7PKI). Residues that are involved in forming salt bridges between RBD and hACE2 are shown as stick structures and are labeled. (**D**) Structural comparison of BtKY72 RBD-laACE2 with hACE2 (PDB: 6LZG). Key contact residues are shown as stick structures and labeled. Salt bridge is colored yellow.

Inspired by the discovery that the strong electrostatic interaction between RBD K493 and hACE2 K31D enables BtKY72 RBD to bind to hACE2, we then investigated whether the original hACE2 K31 could form strong hydrophilic interaction with RBD D493/E493, thereby conferring the binding between the two molecules. As displayed in Fig. 4, two BtKY72 RBD mutants, K493E and K493D, were observed to bind to hACE2 (Fig. 4A). K493E mutant displayed a higher affinity than K493D mutant (22.57 µM vs >128 µM) (Fig. 4A). Therefore, we hypothesized that the BtKY72 RBD mutants that electrostatically interact with hACE2 may confer BtKY72 the hACE2-binding capacity.

To verify this hypothesis, a structural investigation of complexes between sarbecovirus RBDs and hACE2 was performed to identify the residues that contribute to the salt bridge interaction between RBDs and hACE2. The charged residues in RBDs at positions 417 (K417 or R417), 439 (R439), and 493 (K493 or R493) were observed to form salt bridges with hACE2 oppositely charged residues D30, E329, E35, or D38, respectively (Fig. 4B). These residues with addition of residues E493/D493 could be classified into four types, K417/R417, R439, K493/R493, and E493/D493. Because BtKY72 RBD contains residue K493, we only introduced mutations to RBD at positions 417 and 439, including V417K, V417R, and N439R, and tested the binding ability of these mutants to hACE2. Through SPR assay, these three mutants were observed to interact with hACE2, with the BtKY72 RBD V417K mutant showing a superior binding affinity than the RBD V417R mutant (Fig. 4C). These results support our hypothesis.

Although BtKY72 RBD contains K493 and hACE2 includes E35, two residues constituting the salt bridge, no binding between the two molecules was observed. Further structural analysis indicated that the conformation of K493 shifted as observed in the BtKY72 RBD-laACE2 complex provided in this study, probably due to the intrachain salt bridge between BtKY72 RBD K493 and E490 (Fig. 4D). Supportively, BtKY72 RBD E490F mutant (based on SARS-CoV-2 RBD F490), which abolishes the K493-E490 salt bridge, bound to hACE2 with a *K*_D_ of 8.36 µM (Fig. 4E). Moreover, RBD E490F/K493R mutation also enabled BtKY72 RBD to interact with hACE2, although the binding affinity was much low (Fig. 4F).

To evaluate if the observed binding translated into cell entry, we generated VSV particles pseudotyped with the PT BtKY72 or its mutants, which exhibit relatively high affinity for hACE2, including V417K, N439R, E490F, and K493E. These pseudoviruses were then tested for their entry into HEK293T cells expressing hACE2. K493Y/T498W double mutations were reported to enable BtKY72 pseudovirus to enter the cells (34), serving as a positive control. We detected minimal entry of BtKY72 into the cells. However, compared to PT BtKY72, there was an enhanced spike-mediated entry observed for the K493Y/T498W mutant, along with the V417K, N439R, E490F, and K493E mutants (Fig. S2). The results are consistent with the binding results. Overall, the specific mutations at positions 417, 439, and 493 are sufficient for BtKY72 RBDs to acquire hACE2-binding capacity.

### Multiple single-point mutations affect the binding of RBDs with deletion(s) to hACE2

**Fig 5:**
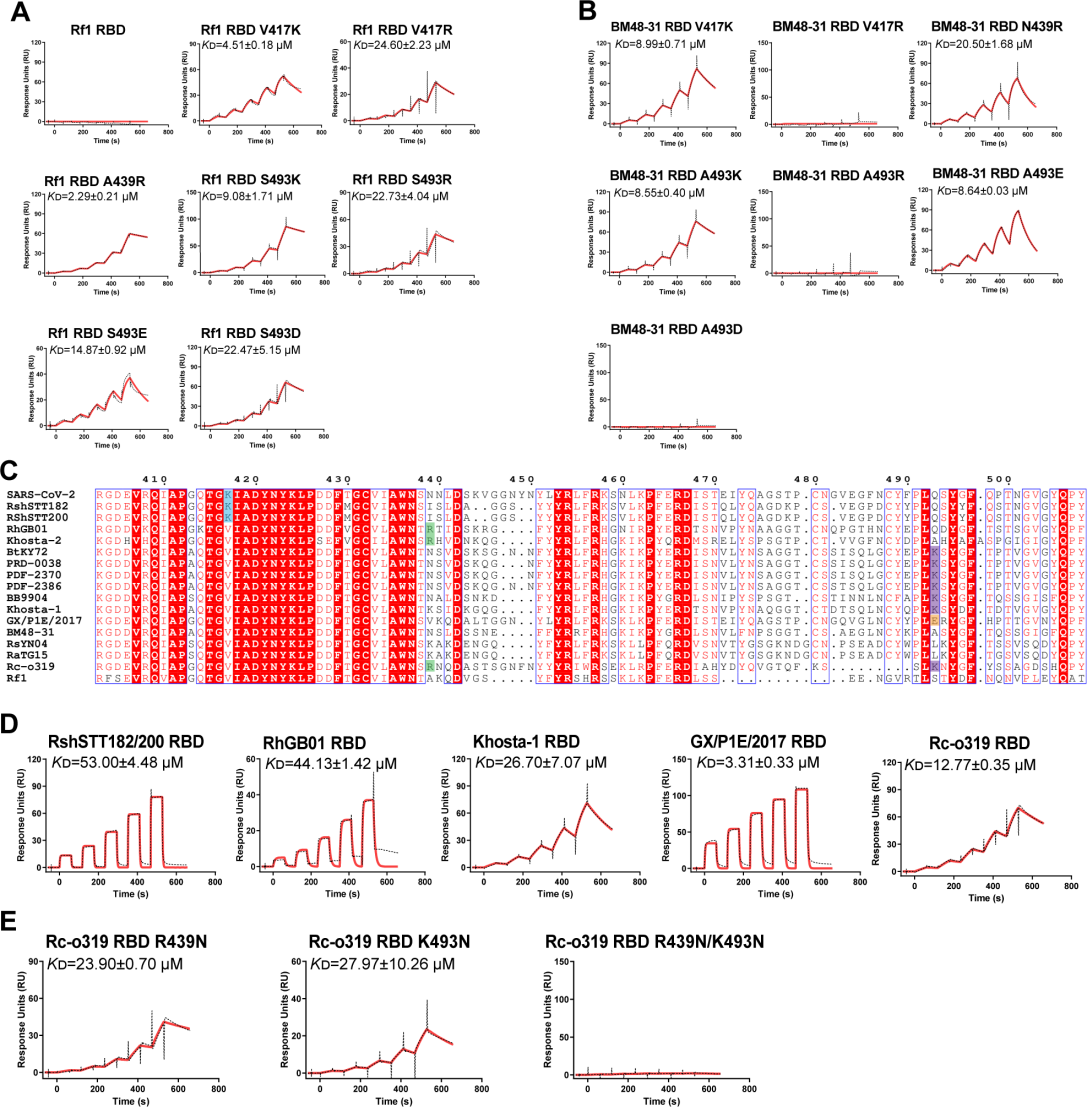
Multiple single-point mutations affect the binding of RBDs with deletion(s) to hACE2. (**A, B, D, and E**) Similar to Fig. 1, hACE2 proteins were captured on a CM5 chip. Rf1 RBD and its mutants (**A**), BM48-31 mutants (**B**), RshSTT182/200, RhGB01, Khosta-1, GX/P1E/2017, and Rc-o319 RBD (**D**), and Rc-o319 RBD mutants (**E**) were used to measure the binding affinity for hACE2. *K*_D_ values are shown in the pictures as means ± SEM of three independent replicates (*n* = 3). (**C**) Sequence alignment of all short-deletion and long-deletion sarbecovirus RBDs, SARS-CoV-2 RBD, and Rf1 RBD proteins. The core residues in sarbecovirus RBDs were labeled by rectangles in different colors.

Considering that the four types of mutated residues are responsible for BtKY72 to obtain the hACE2-binding ability, we referred to these charged residues as core residues. We subsequently explored whether the introduction of the core residues could also confer sarbecoviruses that do not bind to hACE2 the capacity to interact with hACE2. We selected Rf1 (38) as the representative of two-deletion sarbecovirus, which cannot interact with hACE2, along with BM48-31 (Fig. 5A). The mutations, including V417K, V417R, A439R (or N439R), S493K (or A493K), S493R (or A493R), S493E (or A493E), and S493D (or A493D), were introduced to Rf1 (or BM48-31) RBDs one by one and the mutants’ binding to hACE2 were measured. The results showed that the mutations involving each type of core residues could enable Rf1 and BM48-31 RBDs to interact with hACE2 at a micromolar level affinity (Fig. 5A and B). Compared with the mutated K417, K493, and E493, the mutated R417, R493, and D493 have decreased or no effect on the interactions involving Rf1 or BM48-31 RBD with hACE2 (Fig. 5A and B).

Furthermore, we employed pseudovirus infectivity assays to examine the impact of mutations in BM48-31 on entry into hACE2-293T cells. Similar to BtKY72, we selected high-binding mutants, including V417K, N439R, A493K, and A493E, along with the non-binding V417R mutant for testing. The results demonstrated that mutations V417K, N439R, A493K, and A493E promoted the entry of BM48-31 into the cells, whereas V417R did not (Fig. S2), in line with the binding result. This indicates that the core residues may be the common mutated choices for the no hACE2-binding sarbecoviruses to gain the ability to interact with hACE2.

Through sequence alignment, we observed that the most of short-deletion sarbecoviruses and the only member of long-deletion sarbecovirus contain the core residues in their RBDs (Fig. 5C). For example, short-deletion sarbecoviruses RshSTT182/200, RhGB01, Khosta-1, and GX/P1E/2017 has residues K417, R439, K493, and E493, respectively (Fig. 5C). Long-deletion sarbecovirus Rc-o319 contains two core residues: R439 and K493 (Fig. 5C). Next, we probed whether these RBDs bind to hACE2. SPR data revealed that these RBDs exhibited a wide range of binding affinities for hACE2, which varied from 3.31 μM to 53.00 µM (Fig. 5D). To determine whether both residues R439 and K493 of Rc-o319 contribute to the RBD-hACE2 interaction, we prepared Rc-o319 RBD R439N, K493N, and R439N/K493N proteins to test their interaction with hACE2. These two single amino acid substitutions could also bind to hACE2 with a slightly lower affinity than the PT RBD, while the double mutation (R439N/K493N) lacked hACE2-binding ability (Fig. 5E). Taken together, these results indicate that the core residues are important for sarbecovirus RBDs with deletion(s) to interact with hACE2.

### Core amino acid residues expand the ACE2 usage of full-length SARS-CoV-2 RBD

**Fig 6:**
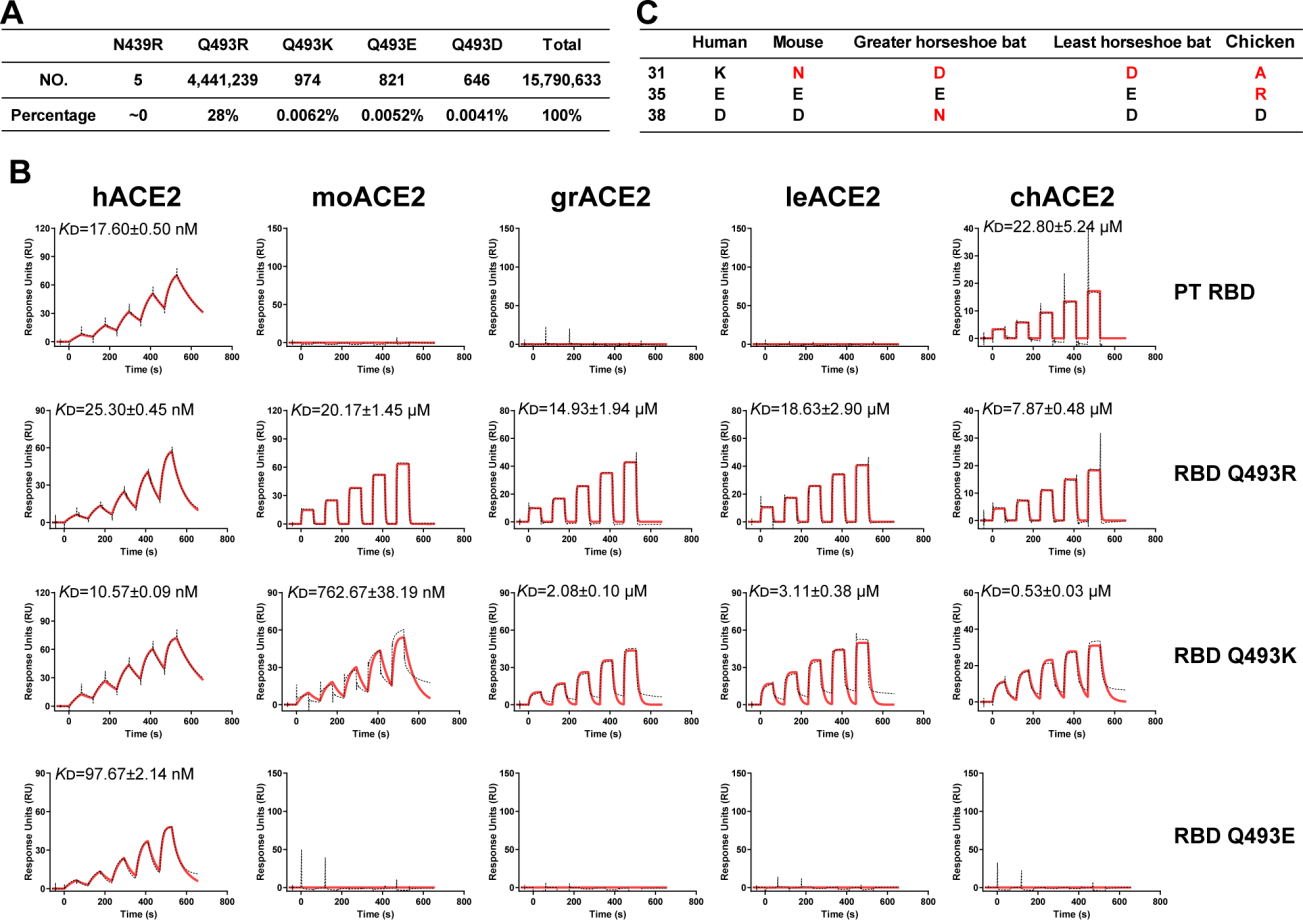
Core amino acid residues expand ACE2 usage of SARS-CoV-2. (**A**) The numbers and percentages of SARS-CoV-2 strains that, respectively, contain N439R, Q493R, Q493K, Q493E, and Q493D were counted from the GISAID database by the end of July 2023. The accession code of the newest strain is EPI_ISL_18001806. (**B**) Similar to Fig. 1, hACE2, moACE2, grACE2, least horseshoe bat ACE2 (leACE2), and chicken ACE2 (chACE2) proteins were captured on the CM5 chip. SARS-CoV-2 RBD and its mutants were used to measure the binding affinity for ACE2s. *K*_D_ values are shown in the pictures as means ± SEM of three independent replicates (*n* = 3). (**C**) Key residues at positions 31, 35, and 38 in ACE2s from humans, mice, greater horseshoe bats, least horseshoe bats, and chickens. The amino acid residues that are different from human ACE2 are colored in red.

We then hypothesized whether core residues would expand the ACE2-binding spectra of the sarbecovirus RBD. The full-length sarbecovirus SARS-CoV-2 PT RBD contains core residue K417. Thus, we focused on the remaining three types of core residues. After searching the GISAID database (gisaid.org), the SARS-CoV-2 strains carrying N439R, Q493K, Q493E, or Q493D mutations were found in very small amounts, while Q493R mutant accounted for about 28% (Fig. 6A). It is due to that Q493R is presented in the Omicron BA.1 and BA.2 subvariants, which were the most prevalent strain in the world. Therefore, we selected Q493R, Q493K, and Q493E mutations and evaluated their functions in the ACE2-binding spectra.

Based on the previous data, PT SARS-CoV-2 cannot bind to certain species’ ACE2s, such as mice, greater horseshoe bats, least horseshoe bats, chickens, etc. (30). Thus, SARS-CoV-2 mutants were evaluated for their binding to the above ACE2 orthologs. Interestingly, SARS-CoV-2 RBD Q493R and Q493K mutations indeed broadened the ACE2-binding spectra, with the ability to bind to mouse, greater horseshoe bat, least horseshoe bat, and chicken ACE2s (Fig. 6B). RBD Q493K displayed higher binding affinities with these ACE2s than RBD Q493R (Fig. 6B), suggesting that it has a superior ACE2-binding adaption. By contrast, the RBD Q493E mutation did not show binding pattern changes. Through sequence analysis, we found these ACE2s contain negatively charged residues at positions 35 or 38, but positively charged residues at positions 31 (Fig. 6C), which could potentially form a salt bridge with R493 or K493, but not E493. Taken together, the newly acquired core residues expanded the ACE2-binding capacity of SARS-CoV-2.

## DISCUSSION

In this study, we found that BtKY72 had a broader ACE2-binding spectrum than another phylogenetic closely relative sarbecovirus, BM48-31. This difference was mainly affected by the RBD residue at position 493 according to the resolved complex structure of BtKY72 RBD bound to laACE2. We further found that four types of core residues affect short-deletion, long-deletion, and two-deletion sarbecoviruses to bind to hACE2, and the core residue K493/R493 expanded the ACE2 usage of SARS-CoV-2. These results highlight the importance of the core residues in sarbecovirus binding to the receptor ACE2. As more sarbecoviruses have been identified as a result of technological development, our data provide the information to predict the threat of the newly emerging sarbecoviruses to humans with more accuracy, which is pivoted for public health pre-warning.

The acquisition of the core residues K493 and R493 expands the ACE2 usage of SARS-CoV-2. Although we did not observe that the core residue E493 confers SARS-CoV-2 the ability to interact with ACE2s from mouse, greater horseshoe bat, least horseshoe bat, and chicken, it cannot be ruled out that SARS-CoV-2 containing Q493E mutation could bind to the ACE2s carrying positively charged residues at position 31. For example, Chinese horseshoe bat ACE2 contains residue K31 and cannot not bind to PT SARS-CoV-2 (30). More research is required to evaluate the interaction between them in the future.

On the other hand, the acquisition of core residues in RBD probably allows more residue substitutions at the interfacial sites while maintaining strong binding to hACE2, thereby conferring more immune escape ability to the mutated strains. For example, the omicron BA.1 sub-variant acquires the core residue R493 (39) but lacks the core residue K417 making it escape from multiple neutralizing monoclonal antibodies (including CB6, P5A-1D2, and P22A-1D1) (40–44) while maintaining a comparable hACE2-binding affinity to the prototype (39). This highlights the superiority of SARS-CoV-2 when it obtains the extra core residues, highlighting the necessity of surveillance of these SARS-CoV-2 variants.

The recent papers showed no binding of BtKY72 RBD to hACE2 using biolayer interferometry or yeast display system (34, 45), and no detectable pseudovirus infection of BtKY72 in the hACE2-293T cells (34). These observations are consistent with our binding results. However, another paper reported that BtKY72 could potentially utilize hACE2 for entry, as observed using a mixed-cell pseudotyped virus infection assay (35). We also observed minimal entry of BtKY72 into the cells. It is possible that additional factors play a role in the cell entry of BtKY72, which will require further detailed analysis in future research.

Horseshoe bats were identified to be the natural hosts of numerous sarbecoviruses. Multiple horseshoe bat ACE2s could efficiently bind to BtKY72, which was found in Kenyan bats in Africa (16, 35). This binding is mainly determined by the salt bridge formed by RBD K493 and ACE2 D31/E31. Thus, there is a need for systematic surveillance of horseshoe bats in Africa, particularly for those that contain residues D31 or E31 in ACE2. With the contact with horseshoe bats, BtKY72 may potentially infect camels or alpacas because of its ability to bind to these two ACE2s. The prevalence of camels and alpacas, especially dromedary camels in Northern Africa (including East Africa) and the deserts of Southwest Asia (46), increases the risk of transmission of BtKY72 or its close relatives globally. It implies that surveillance of coronaviruses in camels is essential.

A limitation of our study was that we focused on the interaction between sarbecovirus RBDs and hACE2. However, it is important to note that the natural hosts for most sarbecoviruses are bats, particularly horseshoe bats. Therefore, further investigations are necessary to gain a better understanding of the binding mechanism of sarbecoviruses to host ACE2s and to assess the viral entry into host cells. This knowledge will be crucial in developing effective pan-epidemic pre-warning measures for the future.

## MATERIALS AND METHODS

### Cells

Expi293F cells (Gibco) were cultured in SMM 293-TII expression medium at 37°C in a shaker incubator (140 rpm). HEK293T (ATCC) cells were cultured in Dulbecco’s modified Eagle medium (DMEM) supplemented with 10% fetal bovine serum (FBS) at 37°C.

### Gene cloning, expression and protein purification

The RBD DNA sequences of BtKY72 (residues 309–530), BM48-31 (residues 310–528), RshSTT200 (residues 306–523), RhGB01 (residues 310–529), Khosta-1 (residues 309–528), GX/P1E/2017 (residues 317–537), Rf1 (residues 310–513), Rc-o319 (residues 293–506), and SARS-CoV-2 (residues 319–541) with N-terminal IL-10 signal peptide sequences and C-terminal hexa-His tag sequences were cloned into the pCAGGS vector (MiaoLingPlasmid). Mutated RBD plasmids were constructed by site-directed mutagenesis using the Mut Express II Fast Mutagenesis Kit V2 (Vazyme). These plasmids were transfected into Expi293F cells using polyethyleneimine (PEI). After 5 days, cell supernatants containing RBD proteins were collected for purification.

The coding sequence of laACE2 (residues 19–615, GenBank: ALJ94034.1) was inserted into the pET21a vector (Novagen). The plasmid was transformed into BL21 (DE3) *E. coli* cells and single colonies were cultured for protein expression at 37°C. Inclusion bodies of laACE2 protein were purified and further refolded in refolding buffer (100 mM Tris-HCl [pH 8.0], 2 mM EDTA, 400 mM L-arginine, 0.5 mM oxidized glutathione, and 5  mM reduced glutathione) by gradual dilution.

The DNA sequences encoding ACE2-mFc from different species were inserted into the pCAGGS vector. The length and accession codes of ACE2 are summarized in Table S2. The plasmids were transiently transfected into HEK293T cells using PEI. Cell supernatants were concentrated for SPR experiments 2 days post-transfection.

The cell supernatants containing RBD protein were passed through a HisTrap HP 5 mL column (Cytiva). The target proteins were eluted with a buffer of 20 mM Tris, pH 8, 150 mM NaCl, and 300 mM imidazole. The RBD proteins and refolded laACE2 protein were further purified by gel-filtration chromatography on a Hiload 16/600 Superdex 200 PG column (Cytiva) in a buffer containing 20 mM Tris, pH 8, 150 mM NaCl. Purified BtKY72 RBD and laACE2 proteins were mixed on the ice overnight and then purified using gel-filtration chromatography on a Hiload 16/600 Superdex 200 PG column in a buffer containing 20 mM Tris, pH 8, 150 mM NaCl. The BtKY72 RBD-laACE2 complex was used for cryo-EM.

### Cryo‐EM sample preparation and data collection

An aliquot of 4 µL purified BtKY72 RBD-laACE2 complex protein (0.32 mg/mL) was subjected to glow‐discharged holey carbon grids (Quantifoil Au 1.2/1.3, 300 mesh). The grids were blotted for 3 s at 100% humidity before flash freezing in liquid ethane using an FEI Vitrobot Mark IV. The data of cryo-EM samples were collected using a Krios electron microscope. The microscope was operated at 300 kV and equipped with a Falcon 4 using AutoEMation. Images were recorded in counting mode with 105,000× nominal magnification, leading to a calibrated pixel size of 0.669 Å. Exposure of 2.53 s was dose‐fractionated into 32 movie frames, resulting in an accumulated dose of 50 e^−^/Å^2^. The defocus range of data sets varied from −1.0 μm to −2.0 µm.

### Image processing and 3D reconstruction

Image drift and anisotropic magnification were corrected using MotionCor2 (47) and initial contrast transfer function (CTF) parameters were estimated using CTFFIND4 (48) Bad images were discarded based on CTF parameters during the initial screening. Particles were subsequently picked using Cryosparc (49). Approximately 3,000 particles were manually picked and subjected to 2D classification to generate templates for further particle auto-picking. After 2D classification, 1,397,414 auto-picked particles were used to generate an initial model in Cryosparc. After several rounds of reference-based 3D classification, the most homogeneous 484,241 particles were selected for the final 3D auto‐refinement. EM data processing is shown in Fig. S3.

### Structure determination and refinement

The crystal structure of the SARS‐CoV‐2 RBD‐hACE2 complex (PDB: 6LZG) was docked into BtKY72 RBD-laACE2 cryo‐EM density maps using UCSF Chimera (50). The atomic model of the structure was built using WinCoot (51) and the refinement was performed using Phenix.refine (52). The stereochemical quality of the final model was assessed using MolProbity (53). Data collection, processing, and refinement statistics are summarized in Table S3. All structural figures were generated using the PyMOL 4.5 software (https://pymol.org/2/).

### SPR assay

Interactions between sarbecovirus RBDs and ACE2 orthologs were tested using an SPR assay on a BIAcore 8K (Cytiva) platform. The assays were performed using a CM5 chip at 25°C in single-cycle mode with PBST system buffer (10 mM Na_2_HPO_4_, 2 mM KH_2_PO_4_, pH 7.4, 137 mM NaCl, 2.7 mM KCl, and 0.005% Tween 20). An anti-mouse IgG antibody (Cytiva) was pre-immobilized on a CM5 chip (Cytiva) via standard amino coupling chemistry to capture soluble ACE2-mFc protein. Serially diluted RBD proteins were used to measure ACE2 binding. After each reaction, the chip was regenerated with glycine (pH 1.7). The equilibrium dissociation constant (*K*_D_) of each interaction was determined using a 1:1 binding model in BIAcore Insight Evaluation 2.0.15.12933 software (Cytiva). All figures were generated using GraphPad Prism version 8.

### VSV pseudovirus entry assays

Pseudoviruses containing the backbone of a deficient vesicular stomatitis virus (VSV) vector (VSV-ΔG-GFP; BrainVTA) and BtKY72, BM48-31 or their mutants S protein were produced following a previously described protocol (37, 54). Briefly, BtKY72, BM48-31, and their mutant S genes with a C-terminal 18-amino-acid deletion were cloned into the pCAGGS vector. HEK293T cells were transfected with 30 µg of the S plasmid in 10 cm dishes. One day post-transfection, cells were infected with VSV-ΔG-GFP pseudoviruses. After 2 h, infected cells were washed with phosphate-buffered saline (PBS) and cultured in media supplemented with anti-VSV-G antibody (produced by I1Hybridoma; ATCC CRL2700). Thirty-five hours post-infection, the supernatants were harvested, concentrated 50× using a 100 MWCO membrane, filtered (0.45-µm filter), aliquoted, and stored at −80°C.

The BtKY72, BM48-31, or their mutant pseudovirus was incubated with 0.5 U/µL BaseMuncher endonuclease (Abcam) at 37°C for 1.5 h to eliminate unpackaged RNA. Viral RNA was then extracted from the treated pseudovirus particles using an RNA extraction kit (Bioer Technology) and quantified using a quantitative reverse transcription-PCR assay with a 7500 Fast real-time PCR system (Applied Biosystems). The primers and probe used to detect the L gene of the VSV virus were as follows: VSV-F, TGATACAGTACAATTATTTTGGGAC; VSV-R, GAGACTTTCTGTTACGGGATCTGG; VSV-probe, 6-carboxyfluorescein-ATGATGCATGATCCWGC-6-carboxytetramethylrhodamine.

BtKY72, BM48-31, and their variants pseudovirus particles were normalized and then diluted to the same amount using quantitative RT-PCR. hACE2-293T cells in 96-well plates were infected with 100 µL of each pseudovirus. After a 15-h incubation, the count of GFP-positive cells was determined using a CQ1 confocal quantitative image cytometer (Yokogawa). Each group contained two replicates.

### Phylogenetic analysis of sarbecovirus RBD sequences

The nucleotide acid sequences of sarbecovirus RBDs were downloaded from the National Center for Biotechnology Information (NCBI; https://www.ncbi.nlm.nih.gov/), GISAID database (gisaid.org), and National Genomics Data Center (China). Accession codes are shown in Table S2. The sequences were aligned using Muscle (codon) in MEGA10 software. The best substitution model (GTR + G + I) for our data set was obtained using MEGA10. The maximum likelihood method was used to construct a phylogenetic tree under the best substitution mode, with 1,000 bootstrap replicates. A phylogenetic tree was generated using iTOL v6 (itol.embl.de).

## Data Availability

The cryo‐EM density maps and corresponding atomic coordinates have been deposited in the Electron Microscopy Data Bank (EMDB) and Protein Data Bank (PDB), respectively. The accession numbers for the BtKY72 RBD-laACE2 cryo‐EM structure in EMDB and PDB are EMD-36892 and 8K4U, respectively. Source data are provided in this paper.

## SUPPLEMENTAL MATERIAL

Supplemental material mbio.01404-24-s0001.docx Supplemental figures and tables.

ASM does not own the copyrights to Supplemental Material that may be linked to, or accessed through, an article. The authors have granted ASM a non-exclusive, world-wide license to publish the Supplemental Material files. Please contact the corresponding author directly for reuse.
