# Supplementary material for: Structural characteristics of BtKY72 RBD bound to bat ACE2 reveal multiple key residues affecting ACE2 usage of sarbecoviruses
Source: mBio. 2024 Jul 31;15(9):e01404-24. doi: 10.1128/mbio.01404-24 (PMC11389363; doi:10.1128/mbio.01404-24)
Supplement: Supplemental material — Supplemental figures and tables. [file mbio.01404-24-s0001.docx]

**Supplementary Information**

**Structural characteristics of BtKY72 RBD bound to bat ACE2 reveal multiple key residues affecting ACE2 usage of sarbecoviruses**

Chao Su^1,2,†^, Juanhua He^1,3,†^, Liang Wang^4^, Yu Hu^1,5^, Jian Cao^1^, Bin Bai^1,^^6^, Jianxun Qi^1^, George Fu Gao^1,2,3,^ ,Mengsu Yang^2,*^, Qihui Wang^1,*^

^†^These authors contributed equally

^*^Corresponding author. Email: [wangqihui@im.ac.cn](mailto:wangqihui@im.ac.cn) (Q.W.); bhmyang@cityu.edu.hk (M.Y.)

**Table S1 Amino acid interactions between BtKY72 RBD and laACE2**

| laACE2 | BtKY72 RBD |
| --- | --- |
| L24 | A476 (2), Y489 (1, 1) |
| T27 | F456 (9), Y489 (10) |
| F28 | Y489 (10) |
| D30 | L455 (2), F456 (2) |
| D31 | F456 (6), Y489 (16), E490 (1), K493 (5) |
| S34 | Y453 (3), L455 (5), K493 (6) |
| A35 | K493 (1) |
| E37 | Y505 (7) |
| N38 | S494 (5, 1), Y495 (2), G496 (6) |
| Y41 | T498 (8, 1), T500 (8, 1), V501 (8) |
| Q42 | T498 (1) |
| L45 | T500 (2) |
| H79 | L486 (3) |
| N82 | L486 (3) |
| N330 | T500 (8) |
| K353 | Y495 (1), G496 (7), F497 (1), V501 (7), G502 (5), Y505 (25) |
| G354 | G502 (7), Y505 (4) |
| D355 | T500 (6), V501 (2), G502 (6) |
| R357 | T500 (4) |
| R393 | Y505 (1) |
| Total | 216 (4) |

The numbers in parentheses of BtKY72 RBD residues represent the numbers of van der Waals contact the indicated residues conferred. The numbers with underline suggest numbers of potential hydrogen bonds between the pairs of residues. van der Waals contact was analyzed at a cutoff of 4.5 Å and hydrogen bonds at a cutoff of 3.5 Å.

**Table S2 Accession code for sarbecovirus RBD and ACE2 sequences**

| RBD | Accession code | RBD | Accession code | ACE2 | Accession code |
| --- | --- | --- | --- | --- | --- |
| SARS-CoV-2 | EPI_ISL_402119^a^ | Rs4237 | KY417147.1 | Human (1-740)^c^ | NP_001358344.1 |
| RaTG13 | MN996532.2 | As6526 | KY417142.1 | Macaca (1-740)^c^ | XP_005593094.1 |
| BANAL-20-52 | MZ937000.1 | FJ2021A | OK017808.1 | Mouse (1-740)^c^ | NP_001123985.1 |
| BANAL-20-103 | MZ937001.1 | Rp3/2004 | DQ071615.1 | Rat (1-740)^c^ | NP_001012006.1 |
| BANAL-20-236 | MZ937003.2 | Rp/Shaanxi2011 | JX993987.1 | Cat (1-740)^c^ | NP_001034545.1 |
| RshSTT182 | EPI_ISL_852604^a^ | RmYN01 | EPI_ISL_412976^a^ | Fox (1-739)^c^ | XP_025842512.1 |
| RshSTT200 | EPI_ISL_852605^a^ | Yunnan2011 | JX993988.1 | Dog (1-739)^c^ | XP_025292925.2 |
| GD/1/2019 | EPI_ISL_410721^a^ | JX2021AC | OK017836.1 | Raccoon dog (1-739)^c^ | ABW16956.1 |
| MP789 | MT121216.1 | HB2020D | OK017801.1 | Pig (1-740)^c^ | XP_020935033.1 |
| SARS-CoV | AY772062.1 | Rs_672/2006 | FJ588686.1 | Civet (1-740)^c^ | Q56NL1.1 |
| Rs3367 | KC881006.1 | Rs4081 | KY417143.1 | Goat (1-739)^c^ | XP_005701129.2 |
| Rs4874 | KY417150.1 | JX2021D | OK017860.1 | Bovine (1-739)^c^ | XP_005228486.1 |
| Rs7327 | KY417151.1 | Rm1/2004 | DQ412043.1 | Rabbit (1-740)^c^ | XP_002719891.1 |
| Rs9401 | KY417152.1 | BtCoV/279/2005 | DQ648857.1 | Sheep (1-740)^c^ | XP_011961657.1 |
| WIV1 | KF367457.1 | GD2019A | OK017825.1 | Horse (1-739)^c^ | XP_001490241.1 |
| WIV16 | KT444582.1 | Longquan-140 | KF294457.1 | Golden hamster (1-740)^c^ | XP_005074266.1 |
| LYRa11 | KF569996.1 | GD2016B | OK017812.1 | Chinese hamster (1-740)^c^ | XP_027288607.1 |
| HKU3 | MT782115.1 | YN2020A | OK017793.1 | Mink (1-740)^c^ | QPL12211 |
| YN2016A | OK017847.1 | GD2017F | OK017792.1 | Lesser hedgehog (1-734)^c^ | XP_004710002.2 |
| YN2016B | OK017848.1 | SC2018B | OK017846.1 | Pangolin (1-740)^c^ | XP_017505752.1 |
| YN2016C | OK017849.1 | AH2021A | OK017807.1 | Camel (1-740)^c^ | XP_006194263.1 |
| YN2016D | OK017850.1 | GZ2021C | OK017829.1 | Alpaca (1-740)^c^ | XP_006212709.1 |
| YN2016E | OK017851.1 | HB2020E | OK017802.1 | Little brown bat (1-742)^c^ | XP_023609439.1 |
| BtRs-YN2018B | MK211376.1 | BtRs-GX2013 | KJ473815.1 | Fulvous fruit bat (1-740)^c^ | ADJ19219.1 |
| RhGB01 | MW719567.1 | BtRs-HuB2013 | KJ473814.1 | Black flying fox (1-740)^c^ | XP_006911709.1 |
| Khosta-2 | MZ190138.1 | BtRl-SC2018 | MK211374.1 | Large flying fox (1-739)^c^ | XP_011361275.1 |
| Rc-o319 | LC556375.1 | BtRs-YN2018A | MK211375.1 | Great roundleaf bat (1-741)^c^ | XP_019522936.1 |
| BtKY72 | KY352407.1 | Rs4247 | KY417148.1 | African yellow bat (1-742)^c^ | QJF77809.1 |
| PRD-0038 | MT726045.1 | HKU3-1 | DQ022305.2 | Brazilian free-tailed bat (1-739)^c^ | QLF98520.1 |
| PDF-2370 | MT726044.1 | HKU3-13 | GQ153548.1 | Greater false vampire bat (1-739)^c^ | QKE49998.1 |
| PDF-2386 | MT726043.1 | RmYN02 | EPI_ISL_412977^a^ | Pallas's mastiff bat (1-739)^c^ | XP_036127492.1 |
| BB9904 | KR559017.1 | RacCS203 | MW251308.1 | Japanese house bat (1-738)^c^ | ACT66266.1 |
| Khosta-1 | MZ190137.1 | BtRs-YN2013 | KJ473816.1 | Greater horseshoe bat (1-740)^c^ | BAH02663.1 |
| YN2020B | OK017852.1 | Anlong-103 | KY770858.1 | Lander's horseshoe bat (1-740)^c^ | ALJ94034.1 |
| YN2020C | OK017853.1 | Anlong-112 | KY770859.1 | Intermediate horseshoe bat (1-739)^c^ | QMQ39222.1 |
| YN2020D | OK017854.1 | F46 | KU973692.1 | Big-eared horseshoe bat (1-740)^c^ | ADN93471.1 |
| YN2020E | OK017855.1 | Rf4092 | KY417145.1 | Pearson's horseshoe bat (1-740)^c^ | ABU54053.1 |
| YN2020F | OK017856.1 | GX2019A | OK017859.1 | Chinese horseshoe bat (1-740)^c^ | ADN93472.1 |
| YN2020G | OK017857.1 | PrC31 | MW703458.1 | Chinese horseshoe bat-1 (1-740)^c^ | QMQ39206.1 |
| YN2020H | OK017858.1 | YN2021 | OK017806.1 | Chinese horseshoe bat-2 (1-740)^c^ | QMQ39216.1 |
| Rs4084 | KY417144.1 | HN2021A | OK017803.1 | Chinese horseshoe bat- 3 (1-740)^c^ | ACT66275.1 |
| RsSHC014 | KC881005.1 | ZXC21 | MG772934.1 | Chinese horseshoe bat-4 (1-740)^c^ | QMQ39200.1 |
| Rs4231 | KY417146.1 | ZC45 | MG772933.1 | Chinese horseshoe bat-5 (1-740)^c^ | QMQ39204.1 |
| GX/P5E/2017 | MT040336.1 | RpYN06 | MZ081381.1 | Chinese horseshoe bat-6 (1-740)^c^ | QMQ39215.1 |
| GX/P5L/2017 | MT040335.1 | LN2020A | OK017794.1 | Chinese horseshoe bat-7 (1-740)^c^ | QMQ39210.1 |
| GX/P4L/2017 | MT040333.1 | BtRf-JL2012 | KJ473811.1 | Chinese horseshoe bat-8 (1-740)^c^ | AGZ48803.1 |
| GX/P2V/2017 | MT072864.1 | JTMC15 | KU182964.1 |  |  |
| GX/P1E/2017 | MT040334.1 | BtRf-HeB2013 | KJ473812.1 |  |  |
| BM48-31 | NC_014470.1 | BtRf-SX2013 | KJ473813.1 |  |  |
| RsYN04 | MZ081380.1 | Jiyuan-84 | KY770860.1 |  |  |
| RaTG15 | CRA004339^b^ | BtCoV/273/2005 | DQ648856.1 |  |  |
| Rf1 | DQ412042.1 | YNLF_31C | KP886808.1 |  |  |
| Rs4255 | KY417149.1 | YNLF_34C | KP886809.1 |  |  |

^a^ Accession code is from the GISAID database; ^b^ Accession code is from National Genomics Data Center (China); Others are from the National Center of Biotechnology Information (NCBI); ^c^ Numbers in parentheses represent ACE2 residues used for protein expression.

**Table S3 Cryo-EM data collection and refinement statistics for** **BtKY72 RBD-laACE2 complex**

|  | BtKY72 RBD-laACE2 |
| --- | --- |
| Data collection |  |
| Magnification | 105K |
| Voltage (kV) | 300 |
| Electron exposure (e^-^/ Å^2^) | 50 |
| Micrographs collected (no.) | 5,176 |
| Defocus range (μm) | -1.0 ~ -2.0 |
| Pixel size (Å) | 0.669 |
| Symmetry imposed | C1 |
| Initial particle images (no.) | 1,397,414 |
| Final particle images (no.) | 484,241 |
| Map resolution (Å) | 3.2 |
| FSC threshold | 0.35 |
| Refinement |  |
| Initial model used (PDB code) | 6LZG |
| Model composition |  |
| Non-hydrogen atoms | 6323 |
| Protein residues | 778 |
| Ligands | 1 |
| *B* factors |  |
| Protein | 60.79 |
| Ligand | 60.13 |
| R.m.s. deviations |  |
| Bond lengths (Å) | 0.003 |
| Bond angles (°) | 0.640 |
| Validation |  |
| MolProbity score | 1.87 |
| Clashscore | 3.72 |
| Poor rotamers (%) | 4.92 |
| Ramachandran plot |  |
| Favored (%) | 96.90 |
| Allowed (%) | 3.10 |
| Outliers (%) | 0.00 |

**Supplementary Figure legends**

**
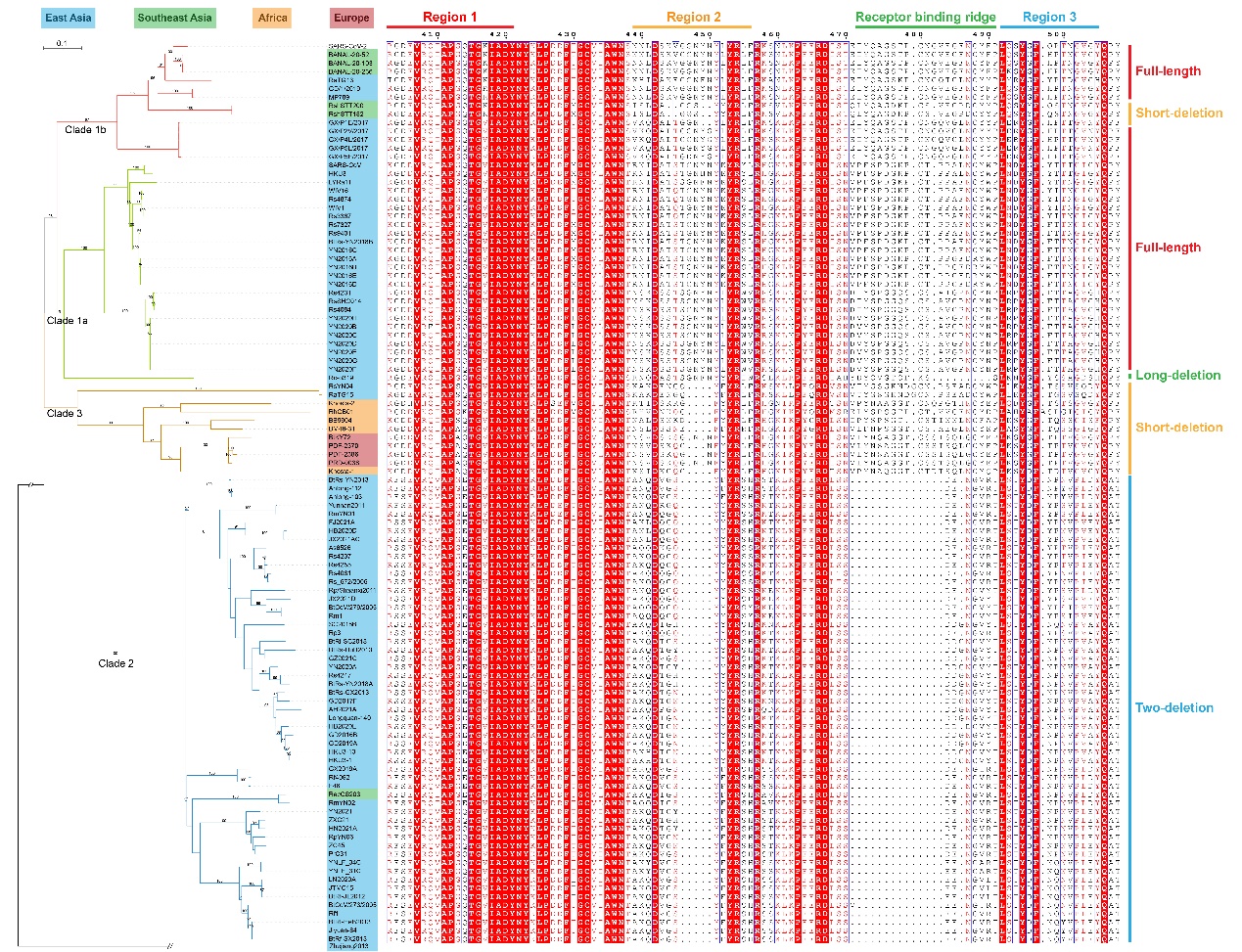
**

**FIG S1 Phylogenetic tree of sarbecovirus RBDs**. Phylogenetic analysis of nucleotide acid sequences of 106 sarbecovirus RBD genes. Number at the nodes indicate bootstrap values in percentage. The colors of branches and rectangles indicate the clades and the locations where the viruses were discovered, respectively. The colored lines on the protein sequences indicate the binding regions in RBD according to the previous report (1). The sarbecovirus with full-length, short-deletion, long-deletion, or two-deletion RBD according to the deletion of regions 2 or receptor binding ridge were labelled on the right.


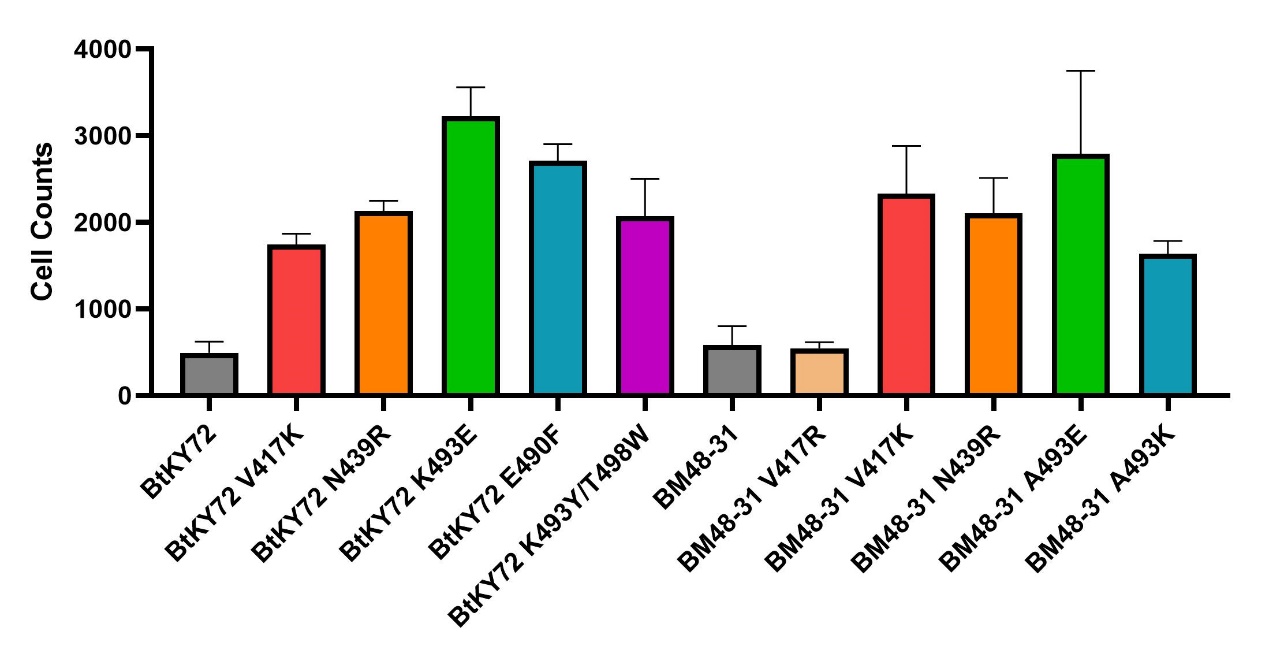


**FIG S2** **Transduction of the pseudotyped BtKY72, BM48-31, and their mutants on 293T-hACE2 cells**. Error bars represent the SD from twice replicates. Pseudovirus infection assays were performed at least twice.


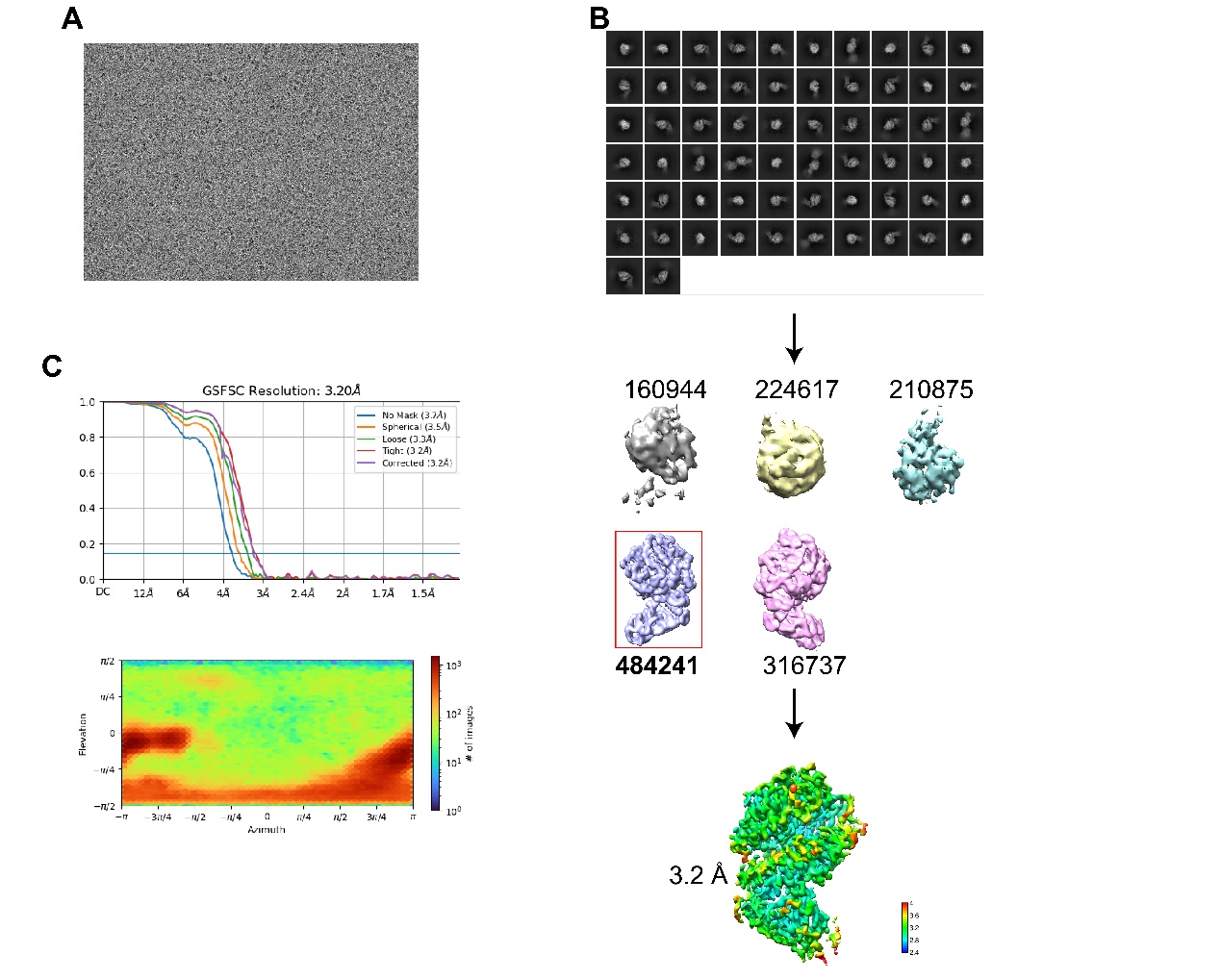


**Fig S3 EM data processing for the BtKY72 RBD-laACE2 complex.** (A) One of the raw cryo-EM micrographs. (B) Schematic to illustrate steps in cryo‐EM data processing. Red rectangle indicates the particles selected for 3D auto-refinement. (C) FSC curve (top) and viewing direction distribution (bottom) of the map.

**References**

1. Wells HL, Letko M, Lasso G, Ssebide B, Nziza J, Byarugaba DK, Navarrete-Macias I, Liang E, Cranfield M, Han BA, Tingley MW, Diuk-Wasser M, Goldstein T, Johnson CK, Mazet JAK, Chandran K, Munster VJ, Gilardi K, Anthony SJ. 2021. The evolutionary history of ACE2 usage within the coronavirus subgenus sarbecovirus. Virus Evol 7:veab007.
